# Relative Entropy of Correct Proximal Policy Optimization Algorithms with Modified Penalty Factor in Complex Environment

**DOI:** 10.3390/e24040440

**Published:** 2022-03-22

**Authors:** Weimin Chen, Kelvin Kian Loong Wong, Sifan Long, Zhili Sun

**Affiliations:** 1 School of Information and Electronics, Hunan City University, Yiyang 413000, China; chenweimin@hncu.edu.cn; 2 School of Computer Science and Engineering, Central South University, Changsha 410075, China; sifan.long@ocibe.com; 3 School of Computer Science, National University of Defense Technology, Changsha 410073, China; 4 5G&6G Innovation Centre, Department of Electrical and Electronic Engineering, Institute for Communication Systems, University of Surrey, Guildford GU2 7XH, UK; z.sun@surrey.ac.uk

**Keywords:** correct proximal policy optimization, approximation theory, reinforcement learning, optimization, policy gradient, entropy

## Abstract

In the field of reinforcement learning, we propose a Correct Proximal Policy Optimization (CPPO) algorithm based on the modified penalty factor *β* and relative entropy in order to solve the robustness and stationarity of traditional algorithms. Firstly, In the process of reinforcement learning, this paper establishes a strategy evaluation mechanism through the policy distribution function. Secondly, the state space function is quantified by introducing entropy, whereby the approximation policy is used to approximate the real policy distribution, and the kernel function estimation and calculation of relative entropy is used to fit the reward function based on complex problem. Finally, through the comparative analysis on the classic test cases, we demonstrated that our proposed algorithm is effective, has a faster convergence speed and better performance than the traditional PPO algorithm, and the measure of the relative entropy can show the differences. In addition, it can more efficiently use the information of complex environment to learn policies. At the same time, not only can our paper explain the rationality of the policy distribution theory, the proposed framework can also balance between iteration steps, computational complexity and convergence speed, and we also introduced an effective measure of performance using the relative entropy concept.

## 1. Introduction

In recent years, artificial intelligence has been successfully applied in many fields of Applied Science. Among them, deep reinforcement learning has made great progress. In particular, AlphaGo, AlphaZero, which are developed by DeepMind, have surpassed the top human players. In practice, OpenAI can even train the same fluent “parkour” action as human beings in robotic control [1]. In addition, deep reinforcement learning also has outstanding performance in games, and exceeds the top human players even in their natural creativity to solve hard problems. Specifically, the core technology that is used by DeepMind is reinforcement learning.

In information theory, entropy is a measure of uncertainty. Entropy is also a commonly used index to measure the uncertainty of random variables in traditional reinforcement learning, which is limited to small action space and sample space that is generally discrete application scenarios. However, more complex and realistic tasks often have a large state space and continuous action space. For example, when the input data are images and sounds, such inputing features often have high dimensions. Traditional reinforcement learning is difficult to deal with. Deep reinforcement learning combines high-dimensional inputting with reinforcement learning. In the field of deep reinforcement learning, the representative research methods are divided into value-based reinforcement learning method, direct policy search-based reinforcement learning method and reverse reinforcement learning method. They have made great breakthroughs in the fields of games, automation control and robotics. For example, Duan et al. used reinforcement learning to control the sustainability of benchmark depth [2]. Qureshi et al. used multi-modal reinforcement learning to train robots in order to acquire social intelligence [3]. Mnih et al. completed the control system that can reach the human level through the reinforcement learning theory [4]. Our contributions to this paper can be divided into the following points:

- From the experimental point of view, the inherent defects of the traditional PPO algorithm are analyzed, and the optimum space is obtained.
- The mathematical model of modified fragments is established to describe the process of reinforcement learning, and the change of probability distribution is used to explain the stationarity analysis of reinforcement learning.
- Through introducing the concept of entropy in information theory, the reward function *H*(*R*) is used to describe the policy distribution function, and *H*(*R*) is quantified by kernel density estimation. The change of *H*(*R*) distribution is evaluated and tested by Kullback-Leibler divergence (as a measure of relative entropy).

The structure of this paper is as follows: Section 2 mainly introduces some important related work in the field of reinforcement learning, some mainstream algorithms, and some derived variant algorithms. Section 3 mainly introduces the traditional PPO algorithm related to the theory of policy distribution, the mathematical model of the evaluation algorithma measure of performance based on the relative entropy concept, as well as the proposed novel CPPO algorithm and related definitions and analysis. Section 4 presents experimental simulations and related results. Section 5 presents conclusions of this paper and is also the future development direction of CPPO algorithm.

## 2. Related Work

### 2.1. Policy Gradient Algorithm

Over the past few years, the policy gradient algorithm has made great progress in deep neural network control, but good results that can be obtained by the policy gradient method are often accompanied with a great cost, because these methods are very sensitive to the numbers of iteration steps: if the selecting step size is too small, the training process will be very slow; if the selecting step size is too large, the feedback signal will be submerged in the noise. The non-convergence of the method may even cause the model to show an avalanche field decline until it collapses [5]. The sampling efficiency of this method is also very low.

Learning simple tasks require of iterations (e.g., millions to billions). Its principle is to control actions randomly and then influence policy changes. Therefore, traditional policy gradient methods cannot avoid the drawbacks of large variance and slow convergence of learning process, such as the representative Deterministic Policy Gradient Algorithms (DPGA) [6]. Based on this motivation, researchers switched to another way of thinking: to find the policy optimization algorithm by variance reduction. The representative algorithms are Trust Region Policy Optimization (TRPO) proposed by Schulman et al. [7]. And Sample Efficient Actor-Critic with Experience Replay (ACER) [8] proposed by Wang et al. Therefore, according to these strategies, Schulman et al. further proposed the Proximal Policy Optimization (PPO) [9,10], which has strong performance and is easy to implement. Although these algorithms have achieved great success, they still have spaces for optimization. For example, because TRPO algorithm is too complex to implement, PPO algorithm is proposed to simplify. In many tasks (e.g., Atari 2600 game), it achieves or even exceeds the performance of TRPO algorithm, and saves a lot of computing resources, so it can be extended to more complex state space fields. Our motivation is also to design more efficient and refined algorithms. 

### 2.2. Proximal Policy Optimization Algorithm

This section mainly introduces some relevant theoretical backgrounds of PPO algorithm, including strategy gradient, advantage function and importance sampling, which paves the way for us to expand the theory of PPO algorithm.

PPO algorithm was proposed by Schulman et al. in order to reduce the complex computational problems that are caused by the TRPO algorithm. Its principle is to estimate the actual strategy (the original strategy distribution of the actual problem) by using the strategy gradient method combined with the stochastic gradient descent algorithm, so how to find the appropriate strategy is the key to solving the practical problem successfully. Generally used strategy gradient estimation can take the following form [9].

(1) $$\hat{g}={\hat{E}}_{t}\left[\nabla_{\theta }\log\pi_{\theta }\left(a_{t}|s_{t}\right){\hat{A}}_{t}\right]$$

where $\pi_{\theta }$ is a random strategy, ${\hat{A}}_{t}$ is an estimate of the advantage function under time step *t*. It can be seen that this estimation method is relatively simple and does not limit the search step size. If the appropriate step size is not selected, there will be a large deviation in the process of strategy updating. In order to solve this problem, the TRPO algorithm used a new proxy objective function to maximize the objective function by searching the confidence domain to approach the original strategy. Therefore, the problem of finding the optimal strategy is transformed into the following constraint problem [7].

(2) $$maximize\;{\hat{E}}_{t}\left[\frac{\pi_{\theta }\left(a_{t}|s_{t}\right)}{\pi_{\theta_{old}}\left(a_{t}|s_{t}\right)}{\hat{A}}_{t}\right]$$

(3) $$\;subject\;to\;{\hat{E}}_{t}\left[KL\left[\pi_{\theta_{old}}\left(\cdot |s_{t}\right),\pi_{\theta }\left(\cdot |s_{t}\right)\right]\right]\leq \delta$$

One advantage of this transformation is that the KL divergence parameter can be used to quantify the distribution differences between the old strategy $\pi_{\theta_{old}}$ and the new strategy $\pi_{\theta }$. As a result, it can be used as a criterion for selecting the appropriate step size. For example, when there are too many differences between the old and new strategies, it reminds us that we should adjust the updating step. Although many approximation methods can be used to simplify the problem for Equation (2), the theory of TRPO algorithm shows that we can use penalty term method to transform into unconstrained problem often has better performance [7].

(4) $$\underset{\theta }{maximize}\;{\hat{E}}_{t}\left[\frac{\pi_{\theta }\left(a_{t}|s_{t}\right)}{\pi_{\theta_{old}}\left(a_{t}|s_{t}\right)}{\hat{A}}_{t}-\beta KL\left[\pi_{\theta_{old}}\left(\cdot |s_{t}\right),\pi_{\theta }\left(\cdot |s_{t}\right)\right]\right]$$

After further simplifying the proxy function of strategy search, the PPO algorithm simplifies the computation of TRPO algorithm by constructing a monotonously increasing strategy function, and gives a more refined form by truncating the proxy target as follows:

(5) $$L^{CLIP}(\theta )={\hat{E}}_{t}\left[min\left(\gamma_{t}(\theta ){\hat{A}}_{t},clip\left(\gamma_{t}(\theta ),1-\varepsilon ,1+\varepsilon \right)\right)\hat{A}\right]$$

where ε is a super parameter (the parameter set at the beginning of the learning process, the empirical value is 0.2). By this truncation method, the step size of strategy updating is controlled within a certain range, so as to prevent the uncontrollable impact on learning caused by the too fast updating process. Another way to optimize the agent’s objective is through the adaptive KL penalty coefficient method, which is not introduced in detail in this paper. Therefore, the pseudo code of the PPO method is shown in Algorithm 1 [9].

The standard solution of TRPO algorithm is that the objective function is approximated by the first order, the constraints are expanded by Taylor’s second order, and then the conjugate gradient method is used to solve the optimal update parameters. However, when the strategy is represented by deep neural network, the standard solution of TRPO algorithm will have a large amount of computation. Because the conjugate gradient method requires the second-order expansion of the constraints, the calculation of the second-order matrix will consume a lot of computational resources. PPO algorithm is the first-order approximation of TRPO algorithm, so it can be applied to large-scale policy updatings, which also explains why the TRPO algorithm in Atari 2600 game can reach or even exceed the performance of PPO. Although PPO algorithm has these excellent performances, there are still some spaces for optimization, so our work starts from the problems of improving PPO algorithm.
**Algorithm 1:** PPO **Input:** Number of iterations episode **Output:** Rewards, losses initialization **for** iteration = 1, 2, 3, …, *M* **do** **while** actor = 1, 2, 3, …, *N* **do**  run policy $\pi_{\theta_{old}}$ for *T* timesteps.  compute advantage estimates ${\hat{A}}_{1}$$,\;{\hat{A}}_{2}$$,\;{\hat{A}}_{3}$$,\;\ldots ,\;{\hat{A}}_{T}$; **end** optimize surrogate L wrt θ; $\theta_{old}\leftarrow \theta$; **end**

### 2.3. Entropy in Information Theory

In the physical world, entropy is a parameter describing the disorder of things. The greater the entropy, the more chaos. Similarly, in information theory, entropy represents the uncertainty of random variables. Given the random variable $X=\left\{x_{1},x_{2},x_{3},\cdots ,x_{m}\right\}$, then the information entropy is

(6) $$H(X)=\sum_{i=1}^{m}p(x_{i})\cdot \log\frac{1}{p(x_{i})}=-\sum_{i=1}^{m}p(x_{i})\cdot \log p(x_{i})$$

Equation (6) shows that information entropy can also be used as a measure of system complexity. If a system is more complex and therefore has more unknown states inside it, its information entropy is larger. Conversely, if a system is simpler, it contains fewer kinds of situations. Under extreme conditions, if there is only one state inside the system, its corresponding probability is 1, so its corresponding information entropy is 0, and then the information entropy is the smallest. In practice, different state spaces and evolutionary processes can be considered as a complex system. Therefore, information entropy can be used to quantify their differences, and an effective mathematical model can be established to study their characteristics.

## 3. Methods

### 3.1. Policy Distribution Theory and Relative Entropy

In this section, we will introduce the theory of policy distribution in complex scenarios and give some basic definitions. Finally, we will give the policy distribution evaluation algorithm based on relative entropy. Therefore, we proposed a modified penalty factor *β* based PPO algorithm, which is named Correct Proximal Policy Optimization (CPPO) algorithm. The test results based on Atari 2600 game showed that CPPO algorithm can achieve the performance of PPO algorithm, the converging process is faster, and the learning process is more stable. Therefore, our work starts from implementing the PPO algorithm.

**Figure 1:**
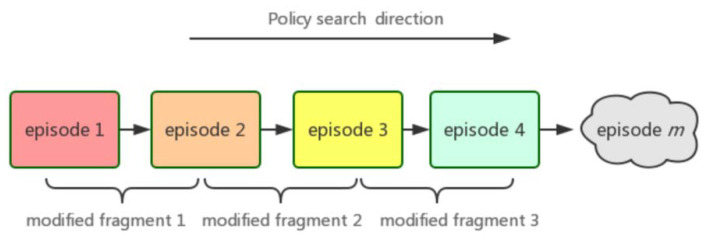
Modified fragment in Markov decision-making process schematic.

**Definition** **1.** *As shown in Figure 1, in Markov decision-making process, each step in the iteration process of policy gradient exploration is recorded as episode, which is represented by symbol ℘. In the state space, we have ℘ = {℘_1_, ℘_2_, ℘_3_, …, ℘n}. For any given ordered fragment [℘_i_, …, ℘_j_ ], and satisfy 0 ≤ i ≤ j ≤ n, we call this fragment as modified fragment, denoted by Ψ, where Ψ = {Ψ1, Ψ2, Ψ3, …, Ψi}*.

**Figure 2:**
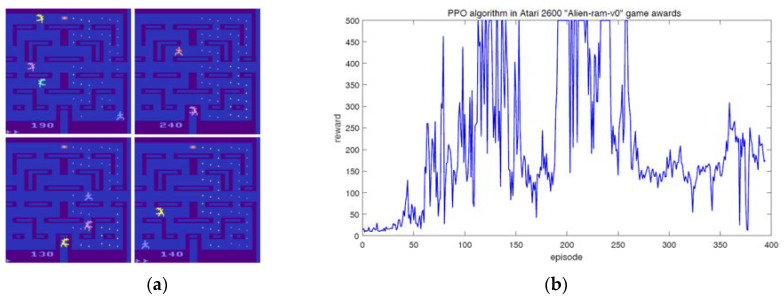
Traditional PPO algorithms in the Atari 2600 game whereby the OpenAI experimental platform is used here, which is a comprehensive experimental platform for testing the new algorithm. (**a**) PPO algorithm in Alien-ram-v0. (**b**) Reward of PPO algorithm in Alien-ram-v0.

Next, we will explain the necessity of defining modified fragments. PPO algorithm can achieve good results in the overall state space (e.g., episode can reach millions or even higher), but there are large fluctuations in some specific small area for update steps. For example, in the ℘∈ (150, 200) ∪ (250, ∞) region of Figure 2b. To solve these problems, we give the reason that when agents explore the state space through random strategies, there may be over-fitting when using proximal policy optimization policy. Therefore, there is such a situation that we can believe is that the sub-distribution of the update agent policy has changed while the overall policy has not changed. The traditional PPO algorithm adapts to this change by truncating the agent target, so that the new policy reaches a certain threshold when old policy changed. The agent target is truncated by mandatory constraints to ensure that the policy update is within a reasonable range. Next, we will show the relationship between episode set and modified fragment.

**Theorem** **1.** *According to Definition 1, we can redefine the policy update process with modified fragment Ψ, In other words, when a task learning process is described as ℘ = {℘_1_, ℘_2_, ℘_3_, …, ℘n}. At the same time, the corresponding modified fragment is described as Ψ = {Ψ_1_, Ψ_2_, Ψ_3_, …, Ψ_i_}, and satisfy I < n. If and only if i = n, episode set is equivalent to modified fragment set. We call it as a sufficient and necessary condition for equivalence*.

**Proof.**  Obviously, *Ψ* is a subset of ℘, in terms of inclusion relations of assemblage, when the [*℘_i_*, …, *℘_j_*] interval of ordered fragments is 0 and satisfy *i* = *n* (℘*_i_* = epsilon *i*). It shows that we have not partitioned the subset, so we derive that ℘ is equivalent to Ψ, therefore, the sufficient condition is proved. When ℘ is equivalent to Ψ, it is easy to know that the [*℘_i_*, …, *℘_j_*] interval of ordered fragments is 0. So, we can conclude that *i = n*, necessary condition is proved. ☐

From the perspective of policy updating, the basic principle of reinforcement learning is through the Markov decision-making process (*S*, *A*, *P*, *R*, *γ*), where *S* is a state set, *A* is the action set, *P* is the state transition probability, *R* is a reward function, *γ* is the discount factor, which are used to calculate cumulative rewards [11], the optimization algorithm is used to approximate the real policy distribution in the actual task, so as to implement efficient decision-making process. Therefore, in this context, we will use the idea of statistical probability distribution to quantify the process.

**Hypothesis** **1.** *For general reinforcement learning tasks, in the process of policy updating, we can assume that the total distribution of policies is π* *(the distribution that we will eventually approximate), and can be divided into $\pi_{1}$* *, $\pi_{2}$* *, $\pi_{3}$* *, …, $\pi_{\theta }$* *, and satisfy $\pi =\pi_{1}⊗\pi_{2}⊗\pi_{3}\ldots ⊗\pi_{\theta }$* *, where the symbol ⊗* *is a merging operation of policy distribution law, which is often unknown in reality.*

Our assumption is mainly based on such a prerequisite, because in the experiment, we found an interesting phenomenon. Take the background of the Atari 2600 game application as an example, we present the following expressions: when the protagonist of the game is close to the enemy, the policy is *π_dangerous_state_*; using *π_security_state_* to express the policy will stay away from the enemy; using *π_center_state_* to indicate the policy used when not approaching or away from the enemy. In a small range, we can think that the policy distribution after these partitions is different when the overall policy distribution is unchanged. For example, when an enemy is close, the correct decision of policy *π_dangerous_state_* is to keep away from the enemy as far as possible. Although the description of policies is abstract, it does not mean that mathematical tools can not be used to analyze them. As a matter of fact, we can use deep neural networks to approximate these policies. Meanwhile, we can use the concept of entropy in information theory to measure the value of policy distribution differences, and use the letter *H* to express the value of entropy [12,13,14,15]. Therefore, through the above examples, we will further refine the general principles in the next section.

### 3.2. Fitting of Reward Function

In order to describe the distribution in modified fragments, we can quantify the difference of modified fragments distribution. For the fitting of policy distribution, we have two methods to fit the modified fragment distribution: parameter estimation method and non-parameter estimation method. The two methods have their own advantages. The parameter estimation rule assumes that the sample set obeys a certain distribution, and then the parameters in the distribution are fitted according to the sample. For example, maximum likelihood estimation and estimation of Gaussian mixtures [16] can be used, but a great deal of prior knowledge of human subjectivity needs to be added. The non-parametric estimation method does not need prior knowledge, but fits the distribution according to the characteristics and properties of the sample itself, so it may be more suitable for the field of reinforcement learning in complex environments. The commonly used non-parametric estimation method is the kernel density estimation method, which can estimate the modified fragment distribution [17].

In modified fragment, according to Hypothesis 1, the distribution it obeys is $\pi =\left\{\pi_{1},\pi_{2},\pi_{3},\ldots ,\pi_{\theta }\right\}$. We need to estimate the distribution obeyed in the modified fragment. The actual policy is very complex. We use deep neural network to approximate π [18]. Because our ultimate motivation is to judge whether the distribution of policies has changed, we use the reward function to approximately replace the impact of the distribution of policies, so we can know whether the distribution of policies has changed or not. That is,

(7) $$E\left[\pi_{1},\pi_{2},\pi_{3},\ldots ,\pi_{\theta }\right]\Rightarrow E\left(R_{1},R_{2},R_{3},\ldots ,R_{\theta }\right)$$

where $\pi_{1},\pi_{2},\pi_{3},\ldots ,\pi_{\theta }$ is a practical policy. $R_{1},R_{2},R_{3},\ldots ,R_{\theta }$ is the corresponding reward function. We use “⇒” symbols to define action operation, which requires that the action object and the object to be acted have a single correlation effect. For example, when one side changes, the other side will change accordingly. It can also be regarded as an extended operation of function operation, which belongs to weak operation, and the action objective function here is a reward function. After the transformation of action operation, we can reduce the complex policy distribution to a relatively simple function. It is much easier to deal with simple functions. In the field of reinforcement learning, the data of reward function is very easy to obtain, so we estimate the reward function *Ri* by kernel density estimation method based on these data.

In the *i*-th modified fragment *Ψ_i_*, we suppose that $R_{1},R_{2},R_{3},\ldots ,R_{\theta }$ is a sample of *R* from the overall reward function. Here, R represents the observed value of the sample, and therefore, in interval *Ψ_i_*, the total density function in any point *r* is

(8) $$\Gamma_{h}\left(r\right)=\frac{1}{nh}\sum_{i=1}^{n}K^{*}[\frac{r-R_{i}}{h}]$$

where $K^{*}\left(\cdot \right)$ is a kernel function, *h* is called window, and in order to satisfy the statistical significance of Equation (8), the kernel function is required to satisfy the

(9) $$K^{*}\left(r\right)\geq 0,\int_{-\infty }^{+\infty }K^{*}\left(r\right)dr=1$$

We can take the Gaussian kernel [19], Laplace kernel [20], or polynomial kernel for commonly used to represent the value of *K*(*r*). Therefore, we derive that in any modified fragment *Ψ*, the distribution function of the reward function Γ(*r*) is estimated by the kernel function. After such transformation, the distribution of the reward function Γ(*r*) can be examined to evaluate the quality of the policy function.

### 3.3. Difference Measurement of Reward Function by Relative Entropy

In the previous section, we have estimated the distribution of the reward function *Γ*(*r*) by using the kernel function method, because each reward function is different among all segments of modified fragment *Ψ*. Kullback-Leibler divergence, an important index widely used in different fields [21,22], is introduced to quantify the difference between policies [21], in this way, the change of *Ψ* distribution can be calculated, which can help us to judge the change of policy distribution.

Firstly, by discretizing the continuous reward function, we introduce the concept of entropy in the reward function *Γ*(*r*) [23]. In the *i*-th fragment, if the reward function is a random variable *R*, then the possible value of *R* is $R^{i}=\left\{R_{1}^{i},R_{2}^{i},R_{3}^{i},\ldots ,R_{\theta }^{i}\right\}$, the corresponding probability distribution is $P^{i}\left(R^{i}=r_{j}^{i}\right)$, where j = 1, 2, 3, …, θ. Then, the random variable *R* of the reward function is defined as:

(10) $$H(R)=-\sum_{i=1}^{n}P(r_{i})logP(r_{i})$$

In machine learning, if the distribution of training data has been fixed, the entropy H (*R*) of the real distribution is a fixed value, therefore, we can use relative entropy to judge the difference between the two distributions, also known as Kullback-Leibler divergence [21,24]. In the *i*-th modified fragment *Ψ_i_* and (*i* + 1)-th modified fragment *Ψ_i_*_+1_. The probability distributions of their reward functions are $\Gamma^{i}\left(r\right)$ and $\Gamma^{i+1}\left(r\right)$. And then the KL divergence of *Ψ_i_* to *Ψ_i_*_+1_ is

(11) $$D\left(\Gamma^{i}∥\Gamma^{i+1}\right)=\int \Gamma^{i}(r)log\frac{\Gamma^{i}(r)}{\Gamma^{i+1}(r)}$$

Relative entropy can measure the distance between two random distributions. When two random distributions are the same, their relative entropy is 0. When the difference between two random distributions increases, their relative entropy will also increase. Therefore, our motivation is to use this property to describe the difference between two adjacent modified fragments *Ψ_i_* and *Ψ_i+_*_1_. If the difference reaches the pre-determined value, we need to adjust the policy updating step size to ensure the step size is within a reasonable range and the stability of the algorithm is maintained in the convergence process. We give a policy distribution evaluation (PDE) as in Algorithm 2.
**Algorithm 2:** PDE **Input:** Policy distribution function **Output**: Policy change outcomes initialization: manual partition of Modified Fragments $\Psi_{1},\Psi_{2},\Psi_{3},\ldots ,\Psi_{\theta }$, setting threshold *D*_0_. **for** iteration = 1, 2, 3, …, *n* **do** update $\Gamma^{i}$ according to Equation (7). update $\Gamma^{i+1}$ according to Equation (7). update $D(\Gamma^{i}||\Gamma^{i+1})$ according to Equation (10). **if**
$D(\Gamma^{i}||\Gamma^{i+1})\rangle D_{0}$ **then**  Output information: The distribution of policies has changed. **else**  Output information: The distribution of policies has not changed. **end** **end**

### 3.4. Correct Proximal Policy Optimization Algorithm

Previously, we have redefined the distribution range of policy by modifying fragments, and evaluated the performance of policy function by reward function. So in this section, we will introduce CPPO algorithm.

From the PPO algorithm, we know that the value of *β* is easily affected. And the updating methods are adjusted in a fixed way, such as optimizing KL-penalized target by a random gradient descent search algorithm [9]:

(12) $$L(\theta )={\hat{E}}_{t}\left[\frac{\pi_{\theta }\left(a_{t}|s_{t}\right)}{\pi_{\theta_{old}}\left(a_{t}|s_{t}\right)}{\hat{A}}_{t}-\beta KL\left[\pi_{\theta_{\mathrm{old}\;}}\left(\cdot |s_{t}\right),\pi_{\theta }\left(\cdot |s_{t}\right)\right]\right]$$

For computation of $d={\hat{E}}_{t}\left[KL\left[\pi_{\theta_{old}}\left(\cdot |s_{t}\right),\pi_{\theta }\left(\cdot |s_{t}\right)\right]\right]$, if $d<\frac{2}{3}d_{\mathrm{targ}}$, the updating method we chose is $\beta_{new}\leftarrow \beta_{new}/2$, if $d>\frac{2}{3}d_{\mathrm{targ}\;}$, the corresponding update method is $\beta_{new}\leftarrow \beta_{new}\times 2$. Therefore, although *β* can be quickly adjusted by the algorithm, its updating method is relatively fixed, and it is not suitable for the use of learning scenarios in complex environments. So, we adjust *β* by using the modified fragment.

For three adjacent modified fragments *Ψ_m_*_−1_, *Ψ_m_*, *Ψ_m+_*_1_, The information entropy of the influence is calculated by Equation (9): $H_{\psi_{m-1}},H_{\psi_{m}},H_{\psi_{m+1}}$. In the updating process, the corresponding penalty factor *β* is *β_m_*_−1_, *β_m_*, *β_m_*_+1_. Therefore, in order to make the algorithm converge more smoothly and increase robustness, we need to ensure that the penalty terms can be reasonably selected. If the *β* is too small, it obviously does not achieve the effect of constraints. If it is too large, the algorithm will produce a lot of shocks, which may eventually lead to poor performance and even difficult to converge. The PDE algorithm can help us find out the difference between the two distributions, but how to quantify the difference is the next problem we need to solve. Therefore, we give the following formula:

(13) $$\xi =H_{\psi_{m-1}}-H_{\psi_{m}}$$

According to Hypothesis 1, at the beginning of distribution change, policy updates are often greatly affected. When *β* is modified, the condition to be satisfied is |*ξ*| ≥ *ξ*_0_, where *ξ*_0_ is our pre-set threshold, and satisfies the condition *ξ* ≥ 0, according to the Equation (13) and principle of entropy, there are two different cases.

**Case** **1.** *When the condition ξ > ξ_0_ is satisfied, this indicates that the distribution of the modified fragments Ψ_m−1_ and Ψ_m_ has changed significantly, therefore, the update policy should slow down the step size*.

**Case** **2.** *When the condition ξ < ξ_0_ is satisfied, this indicates that the distribution of the modified fragments Ψ_m−1_ and Ψ_m_ has changed slightly, so the update policy should increase the step size*.

We adjust *β* by using the following modification functions. Assuming that the output of PED algorithm is true or false, where true represents a change in the policy distribution, we need to adjust the updated parameters, false represents no change in the policy distribution, we can maintain the current update parameters. For the case of true, we use the principle of entropy to modify the value of *β* by using the correction function Υ, that is $\Upsilon \left(\beta ,H_{\psi_{m-1}},H_{\psi_{m}}\right)$, the correction function Υ can be used in the following expression when it is reduced:

(14) $$\gamma \left(\beta ,H_{\psi_{m-1}},H_{\psi_{m}}\right)=\beta *\left[\left|\frac{\min\left(H_{\psi_{m-1}},H_{\psi_{m}}\right)}{\max\left(H_{\psi_{m-1}},H_{\psi_{m}}\right)}\right|\right]$$

Therefore, through the above deduction, one assumes that when the distribution changes and according to the results of Equation (12), we can adjust *β* according to the following Equation (15).

(15) $$\beta_{m+1}=\{\begin{array}{l} \beta_{m}^{ppo}*\left[\frac{\min\left(H_{\psi_{m-1}},H_{\psi_{m}}\right)}{\max\left(H_{\psi_{m-1}},H_{\psi_{m}}\right)}\right],\xi >0\\ \beta_{m}^{ppo}*[\frac{\max\left(H_{\psi_{m-1}},H_{\psi_{m}}\right)}{\min\left(H_{\psi_{m-1}},H_{\psi_{m}}\right)}],\xi <0 \end{array}$$

where $H_{\psi_{m-1}},H_{\psi_{m}},H_{\psi_{m+1}}$ is the corresponding entropy of the extracted fragment. $\beta_{m}^{ppo}$ is the old value of *β* in PPO algorithm before the next update. We give the pseudo code of CPPO algorithm as shown in Algorithm 3.
**Algorithm 3:** CPPO initialization: Calculation of $\beta_{1},\beta_{2},\beta_{3}$ by PPO algorithm, state; classification of $\psi_{1},\psi_{2},\psi_{3},\ldots ,\psi_{\theta }$; **for** *j* = 1, 2, 3, …, *n* **do** execute algorithm 1 and return the value of state; **if** *state==true* **then**  //Updating *β* according to Equation (14);  $\beta_{j+1}^{cppo}=\Upsilon \left(\beta_{j}^{ppo},H_{\psi_{m?1}},H_{\psi_{m}}\right)$ **else**  $\beta_{j+1}^{ppo}\leftarrow \beta_{j+1}^{cppo}$; **end** **end**

Therefore, compared with the traditional PPO algorithm, the corrected CPPO algorithm can search the policy function more accurately. Note that the *β* punishment can more efficiently adjust the inappropriate step size, which in turn has a widespread impact on search results. In the field of optimization, the choice of parameters is often very important, which will have a huge impact on the results.

## 4. Experimental Simulation and Results

In this section, we will verify some difficult problems existing in the traditional PPO algorithm. At the same time, we use the Atari 2600 game to test the algorithm given in this paper and make a comparative analysis with the traditional PPO algorithm. The environment we use is the Intelligent Reinforcement Learning Experimental Environment developed by OpenAI (https://gym.OpenAI.com/envs/, accessed on 11 March 2022). The CPPO algorithm and the PPO algorithm are compared on Atari 2600 game “Alien-ram-v0”, “Asterix-v0”, “Enduro-v0”, “SpaceInvader-ram-v0”. Finally, we make the corresponding return function curve and the corresponding learning cost chart.

Although PPO algorithm is the first-order approximation result of TRPO algorithm, which greatly reduces the computational complexity and can be used in most learning scenarios. Although it can be one of the most popular algorithms in the field of reinforcement learning, it still has some shortcomings. Taking the “Alien-ram-v0” game in Atari 2600 as an example, Figure 2a shows the performance of PPO algorithm in the game, and Figure 2b shows the curve of the corresponding re-ward changing with time step. We can see that in a small range of intervals, the reward can not increase steadily. In other words, the convergence and stationarity of the algorithm still have some optimization spaces.

Figure 2 shows that PPO algorithm can learn the rules of the game quickly by online learning, but as the number of learning cycles (episodes) increases gradually, some unstable factors begin to appear in the proxy target. Therefore, we need to establish the proxy target to suffer from the negative impact of excessive change of policy gradient. At the same time, we want to ensure the robustness and convergence of the algorithm. Hence, we need to find a more stable and robust proxy target.

**Figure 3:**
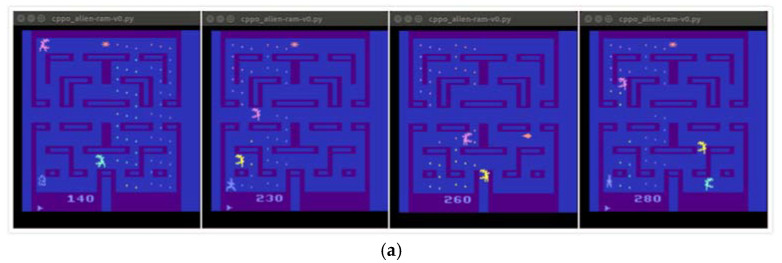

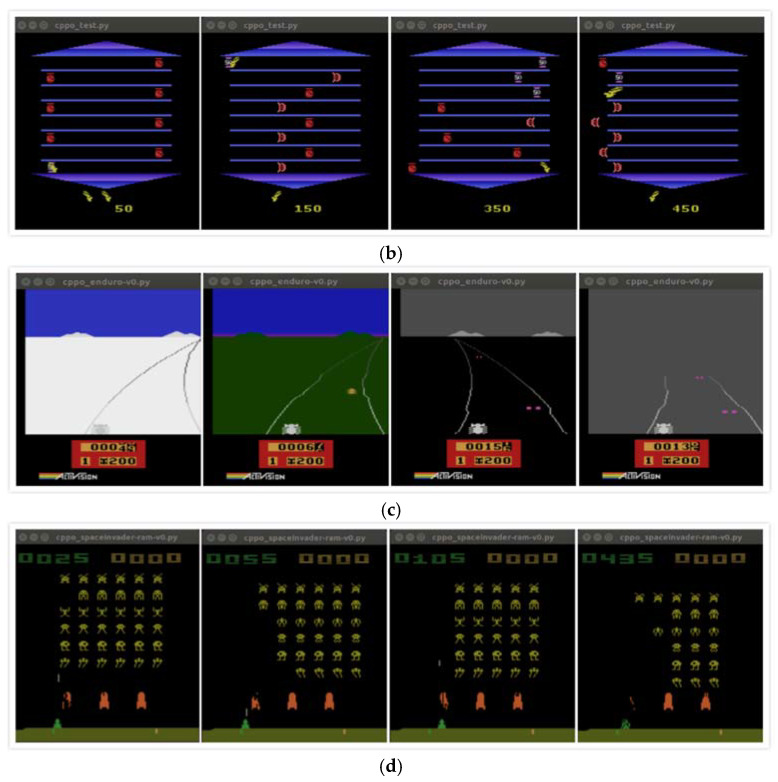
Implementing effect of CPPO algorithm on the Atari 2600 game in the initial stage. (**a**) Alien-ram-v0. (**b**) Asterix-v0. (**c**) Enduro-v0. (**d**) SpaceInvader-ram-v0.

**Figure 4:**
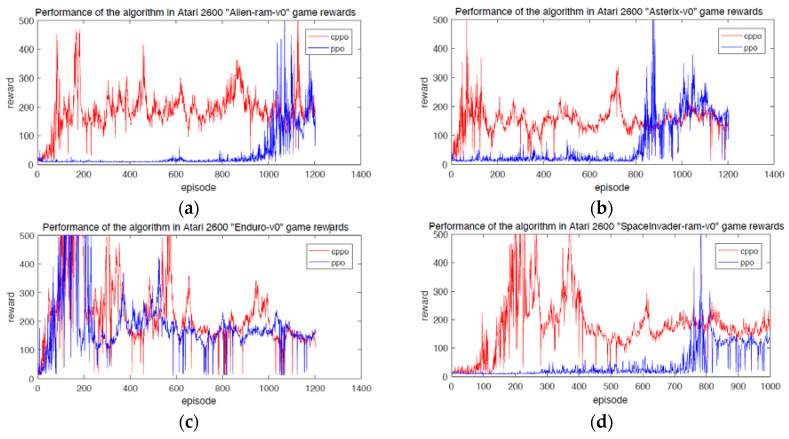
Performance of the algorithm in the Atari 2600 game rewards. (**a**) Alien-ram-v0. (**b**) Asterix-v0. (**c**) Enduro-v0. (**d**) SpaceInvader-ram-v0.

Through the above comparative analysis, the traditional PPO algorithm has a slow convergence speed in the field of complex learning scenarios, so the modified CPPO algorithm proposed in this paper has a strong convergence. It can quickly converge to better results in the initial training period, such as Figure 3a–d. They achieve the performance of PPO algorithm in a very short training period and can maintain a smooth learning process. In addition, Figure 4 shows that CPPO algorithm converges faster than PPO algorithm at the beginning of training, although there is little difference between the convergence of CPPO algorithm and that of the Enduro-v0 game. For different application scenarios [25], the performance may also depend on the step size. According to Hypothesis 1, if the application background is very different, then the step size adjustment and correction will face great challenges. If the KL divergence does not reflect the distribution change well in our modified fragments, then we will make the exploration step size according to the environment scenario. It is difficult to adjust appropriately. This also shows the application scope of CPPO algorithm. In particular, it is suitable for all reinforcement learning tasks and scenarios with obvious changes in distribution.

**Figure 5:**
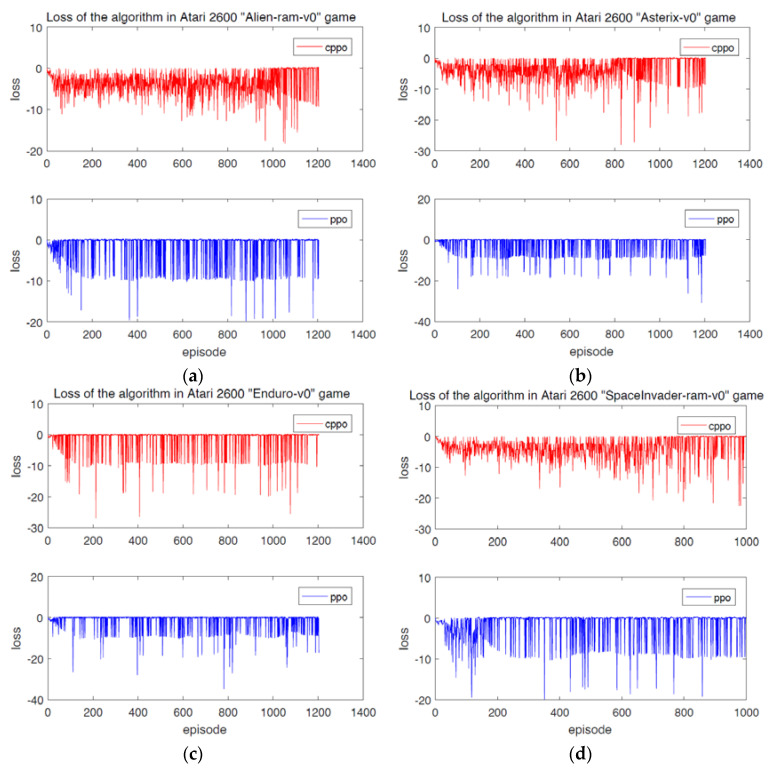
Loss of the algorithm in the Atari 2600 game demonstrating how the CPPO algorithm and PPO algorithm can lose with every iteration of time step. (**a**) Alien−ram−v0. (**b**) Asterix−v0. (**c**) Enduro−v0. (**d**) SpaceInvader−ram−v0.

For supervised learning, the processing strategy of hybrid data to identify useful information and eliminate noise might be explored as much as possible [26,27,28]. However, unlike supervised learning, reinforcement learning derives from experience rather than data set. At this time, exploring and improving the state of reward and acquisition are the target, which means the reward gained by the algorithm in the iteration process is an important index to evaluate the performance of reinforcement learning algorithm. In addition, the loss suffered by the algorithm is also an important index. Figure 5 shows the loss of CPPO algorithm and PPO algorithm on Atari 2600. The total loss of CPPO algorithm proposed in this paper is lower than that of PPO algorithm. Table 1 is a comparison of the total rewards obtained by CPPO algorithm and PPO algorithm in the same number of iterations. As it can be seen, the total rewards of CPPO algorithm are 226,214, 183,496, 267,548 and 175,857, which are much higher than that of PPO algorithm. In addition, the empirical values of *D*_0_ for Equation (7) and ξ_0_ for PDE algorithms are 7.45 and 16.93 respectively. These two parameters will affect the convergence of the algorithm, but the mechanism is not clear yet.

## 5. Conclusions

This paper presents a CPPO algorithm based on the fragment method and relative entropy, which the traditional PPO algorithm. Algorithms in the field of deep reinforcement learning have some inherent shortcomings, such as slow convergence and long training time.

Specifically, the relative entropy that is introduced in this paper is used to quantify the modified fragments, and finally the CPPO algorithm is established. The CPPO algorithm has faster convergence than traditional PPO algorithm, and the focus of after this study is how to further improve the anti-jamming performance algorithm. By reducing the instability caused by random gradient search in PPO algorithm, the instability factor can be.

## Figures and Tables

**Table 1:** CPPO and PPO algorithm achieving total reward based on the same number of iterations.

Performance Game Items	Alien−ram−v0	Asterix−v0	Enduro−v0	SpaceInvader−ram−v0
CPPO	226,214	183,496	267,548	175,857
PPO	45,931	81,578	221,451	43,571
Number of iterations	1200	1200	1200	1000
Iteration time	≥6.5 h	≥7 h	≥7.5 h	≤5 h

## Data Availability

Not applicable.
